# Correlation of periodontal and microbiological evaluations, with serum levels of estradiol and progesterone, during different trimesters of gestation

**DOI:** 10.1038/s41598-019-48288-w

**Published:** 2019-08-13

**Authors:** Renata Santos de Souza Massoni, Andreza Maria Fábio Aranha, Fernanda Zanol Matos, Orlando Aguirre Guedes, Álvaro Henrique Borges, Monize Miotto, Alessandra Nogueira Porto

**Affiliations:** 1 Universidade de Cuiabá, Gynecology and Obstetrics Department, Cuiabá, MT CEP 78065-443 Brazil; 2 Universidade de Cuiabá, Dentistry Department, Cuiabá, MT CEP 78065-443 Brazil

**Keywords:** Biofilms, Dental biofilms

## Abstract

Our purpouse was to identify quantitatively and qualitatively the subgingival flora in different gestational trimesters, compared to non-pregnant women; evaluating the correlations between epidemiological characteristics, clinical diagnosis, microbiological findings and levels of estradiol and progesterone. 52 pregnant women divided into 3 groups, according to the gestational trimester and 15 non-pregnant patients, without hormonal contraceptives, were evaluated. Plaque index (PI), gingival index (GI), probing depth (PD) and clinical attachment level (CAL) were evaluated. Subgingival biofilm samples were processed by the qPCR technique and the serum levels of estradiol and progesterone quantified by chemiluminescence. Clinical diagnosis during gestation was correlated with the total bacterial count. A higher prevalence of *Tannerella forsythia* (*Tf*) was identified in first trimester of pregnancy and this periodontopathogen was correlated with the diagnosis of gingivitis among pregnant women. *Porphyromonas gingivalis* (*Pg*) showed a positive correlation with progesterone levels in the first trimester. High prevalence of periodontopathogens was noticed in this population. Clinical diagnosis in gestation was positively correlated with the total amount of bacteria, without influence of the hormonal levels or the epidemiological factors evaluated. The presence of *Tf* favored occurrence of gingivitis during pregnancy and the progesterone levels in the first trimester enhanced the growth of *Pg*.

## Introduction

The World Health Organization (WHO), through its Global Oral Health Program, highlighted the importance of oral health as a determining factor for individuals to have a good quality of life^1^. Regular professional evaluations and hygiene are key components to achieve good oral health, and it has been established that such care is safe, including during pregnancy, and should be recommended to improve the overall oral health of women^2^.

Although routine dental care for women is performed infrequently during the gestational period, it is clinically noticed that the physiological changes that occur during pregnancy affect oral health and in 1999, the American Academy of Periodontology already listed at least two conditions related to this period: pregnancy-associated gingivitis and pregnancy-associated pyogenic granuloma^3^.

A gradual increase of gingivitis during this period has been observed in several studies, and the role of increasing levels of sex steroids on these clinical manifestations and bacterial biofilms has been questioned^4–8^. In pregnancy, placenta unit progressively increases the production of steroid hormones over the three gestational trimesters, raising estrogen levels to 1000 times above non gestational levels, while progesterone reaches values 10 times higher than non-gestational levels a few weeks before delivery^9^.

The literature describes at least four mechanisms that may contribute to the exacerbation of gingival inflammation in the presence of high levels of estradiol and progesterone^6^. The first is the vasodilatory effect of estrogens, which increase the blood supply to the gingival tissue with a consequent exacerbation of the inflammatory response. In addition, suppression of the immune system, quantitative and qualitative alterations of the supra and subgingival flora, and phenotypic alterations of the gingiva are also indicated^4,6,10,11^. On the other hand, it has been documented that inadequate control of oral biofilms, which is associated with the evolutionary characteristics of biofilms, has a tremendous impact on the progression of oral diseases^12,13^

During biofilm development initially glycoproteins and salivary antibodies create a conditioning film on the dental surface that allows the selective adhesion of the initial colonizing bacteria of the *Streptococcus* group (*S. oralis, S. sanguis, S. mitis and S. gordonii*)^14^. Six microbial complexes that progressively settle in the subgingival biofilm during the natural history of periodontal disease evolution were described^12,13^. After the establishment of the gingivitis, it was documented that the species of red (*Porphyromonas gingivalis, Tannerella forsythia and Treponema denticola*) and orange complexes are more prevalent and found in greater number^15^. It was observed that *Porphyromonas gingivalis* is one of the main etiological agents of chronic periodontitis^16–18^, while *Aggregatibacter actinomycetemcomitans* was present in aggressive periodontitis in 72% of cases^19^.

The purpose of the present study is to investigate clinical periodontal conditions, subgingival biofilm and hormone levels of pregnant women at different trimesters of pregnancy. The null hypothesis of this study was that hormone levels at different trimesters of pregnancy did not affect the periodontal profile and subgingival biofilm.

## Methods

We performed a cross-sectional case control study, with a convenience sample, where sixty-seven women from the Gynecology and Prenatal outpatient clinics of the SUS in Cuiabá, MT, Brazil, were evaluated between February and August 2017.

Inclusion criteria were defined as healthy women between the age of 18 and 35 years with normal singleton pregnancy confirmed by ultrasonography at time of sampling. The control group consisted of healthy non-pregnant patients in the same age group with regular menstrual cycles who did not use a hormonal contraceptive method in the 3 months prior to the study.

Exclusion criteria included: 1) high-risk pregnancies according to the criteria of the Brazil Health Ministry and those of the National Institutes Of Health (NIH), covering pregnant women with comorbidities, obesity, multiple gestations and women who were very young (<15 years old) or much older (>35 years old) (14,15); 2) use of systemic antibiotics in the last four weeks; 3) users of corticosteroids and heparin; and 4) patients with a limited oral opening. Patients under 18 years old were also excluded because they required authorization from third parties to engage in the project.

Each group had different patients. Pregnant women were divided into 3 groups according to gestational age, as listed below. Non-pregnant women were allocated in the fourth group: Group 1 (n = 16) - first trimester of gestation (up to 98 days); Group 2 (n = 21) - second trimester of gestation (between 99 and 196 days); Group 3 (n = 15) - third trimester of gestation (from 197 days); Group 4 (n = 15) - non-pregnant women (control group). All patients were interviewed, then underwent periodontal clinical examination and blood collection for hormonal assessment only once.

A questionnaire was used to collect socioeconomic, obstetric and gynecological data. The study population underwent the application of a Brazilian socioeconomic questionnaire (Brazilian Association of Research Firms) that classify the population into six strata (A = US $ 6480.00, B1 = US $ 4280.00, B2 = US $ 2780.00, C1 = US $ 1420, 00, C2 US $ 780.00, D = US $ 465.00, E = US $ 205.00).

The periodontal examination was performed with the help of a clinical mirror (SSWhite Duflex®, Rio de Janeiro, RJ, Brazil) and a Williams-type periodontal probe (Hu-Friedy® Mfg Co Inc. Chicago, IL, USA), with measurements reported in millimeters.

The clinical measurements analyzed included visible plaque index (PI), gingival bleeding index (GI), probing depth (PD) and clinical attachment level (CAL) which were evaluated for all teeth in the oral cavity. The PI is a dichotomous parameter that considers the presence or absence of visible biofilm on all faces of the teeth, evaluating the patient’s plaque control ability. The GI evaluation occurred after the insertion of a periodontal probe (marked in millimeters) approximately 0.5 mm inside the gingival sulcus, crossing through the buccal and palatal surfaces, waiting 30 seconds to verify the occurrence of bleeding in the marginal gingiva^20^.

For PD evaluation, the distance between the gingival margin and the most apical portion of the junctional epithelium was measured. The CAL was evaluated as the distance between the cementoenamel junction and the most apical portion of the junctional epithelium. The periodontal diagnosis was established after oral evaluation and was classified as healthy (when <30% of the periodontal sites presented gingival bleeding), gingivitis (>30% of the periodontal sites presented gingival bleeding) or periodontitis (presence of four or more teeth with one or more sites presenting PD ≥4 mm and loss of clinical insertion ≥3 mm at the same site)^3^.

Serum estradiol and progesterone levels were estimated by chemiluminescence using Access Estradiol and Progesterone assays (Access Immunoassay Systems, Beckman Coulter, Inc. 250 S. Kraemer Blvd. Brea, CA, USA). For the nonpregnant women, samples were collected between the 7th and 13th days of the follicular phase of the menstrual cycle. For pregnant women, a blood aliquot was collected during the routine exams.

Subgingival microbiological collections were performed after clinical evaluation with the aid of an autoclaved No. 30 paper cone (Dentsply, Maillefer, Ballaigues, Switzerland). Intrasulcular microbial samples were collected from the mesiobuccal sites of teeth 11, 16, 26, 31, 36 and 46. In the absence of these elements, samples of the adjacent teeth were obtained. The paper cone was inserted into the most apical portion of the periodontal sulcus and held in place for 60 seconds^19^. Thereafter, paper cones from each individual were placed in individual Eppendorf minitubes (Bio-Rad®, Hercules, CA, USA) and maintained at a temperature of −80 °C until processed.

Genomic DNA extraction was performed using a PureLinkTM Genomic DNA Purification Kit (Invitrogen, Carlsbad, CA, USA) following the manufacturer’s instructions.

The absolute identification and quantification of *Aggregatibacter actinomycetemcomitans* (Aa), *Porphyromonas gingivalis* (Pg), *Tannerella forsythia* (Tf), *Streptococcus oralis* (So) and Universal (Un) in the clinical samples was performed by qPCR with a StepOne™ instrument (Applied Biosystems, Foster City, CA, USA) using specific primer pairs and amplification with TAQMAN® probes (Applied Biosystems). The primers were tested for specificity using the NCBI BLAST program (http://blast.ncbi.nlm.nih.gov/Blast.cgi). A negative control was performed by replacing the DNA with the same amount of sterile water to check for possible contamination.

The clinical and microbiological data were analyzed using Statistical Package for Social Sciences (IBM Corp. Released 2011. IBM SPSS Statistics for Windows Version 20.0, Armonk, NY: IBM Corp.).

Each clinical parameter was determined by two blinded and previously calibrated examiners according to the methodology described by Araujo *et al*.^21^. For the continuous variables (depth of probing), the SEM (standard error of the measurement) was used, and the Kappa test was used for the categorical variables (plaque and gingival indices). Thus, 10 tests were repeated within 30 days and submitted to analysis. The examiners were considered to be calibrated by SEM ≤ 0.8 and K > 0.8 and <0.95.

To meet the objectives of the study, in addition to performing basic exploratory analysis techniques, such as determining the mean, median, standard deviation, absolute and relative frequency, the Chi-square test was used to analyze the relationship between qualitative variables, and Tukey’s multiple comparison was used to compare groups in pairs. To compare quantitative variables, Pearson’s correlation was used. Finally, to compare means between qualitative covariates, we used the ANOVA test.

The hypothesis tests developed in this study considered a significance of 5%, and the null hypothesis was rejected when the p-value was less than or equal to 0.05.

### Ethical approval

All procedures performed in studies involving human participants were in accordance with the ethical standards of the institutional and/or national research committee and with the 1964 Helsinki declaration and its later amendments or comparable ethical standards. The Ethics committee of the University of Cuiabá issued its approval for this study through opinion number n° 1.898.399.

### Informed consent

Informed consent was obtained from all individual participants included in the study.

## Results

The mean age of participants was 24.67 ± 1.26 years, with no difference between groups (p = 0.704). Regarding epidemiological data, including race, parity and socioeconomic class, the groups were homogeneous (Table 1).

**Table 1:** Description of epidemiological data - absolute and relative frequency, mean and standard deviation (Chi-square test, p < 0.05).

Epidemiological Data			Groups				Total	p-value
			1	2	3	4		
**Marital status**	**With partner**	N (%)	8 (50)	18 (86)	12 (80)	8 (53)	46 (69)	0.047*
**Race**	**Caucasian**	N (%)	6 (38)	4 (19)	6 (40)	4 (27)	20 (30)	0.502
**Parity**	**Primigravida**	N (%)	6 (38)	7 (33)	5 (33)	7 (47)	25 (37)	0.970
**SE** ^**a**^ **class**	**Class E**	N (%)	3 (19)	5 (24)	4 (27)	1 (7)	13 (19)	0.527
	**Class D**	N (%)	13 (81)	14 (67)	9 (60)	12 (80)	48 (72)	
	**Class C2**	N (%)	0 (0)	2 (10)	2 (13)	1 (7)	5 (7)	
	**Class B2**	N (%)	0 (0)	0 (0)	0 (0)	1 (7)	1 (1)	
**Mean age (years)Standard deviation**			23.5+/− 5.6	24.9 +/− 5.0	25.7+/− 4.9	24.5+/− 6.0	24.7+/− 5.3	0.704
**Mean gestational age (days)Standard deviation**			77+/− 14.2	157,48+/− 28.5	236,13+/− 26.8	—	—	—

^a^SE = socioeconomic.

*Significative p-value.

There was small variability in gestational age within each group of pregnant women. Only observed difference was in relation to the marital status, with a higher prevalence of patients with a partner in groups 2 and 3 (p = 0.047). It was observed that 91% of the investigated women belonged to classes D and E.

**Figure 1:**
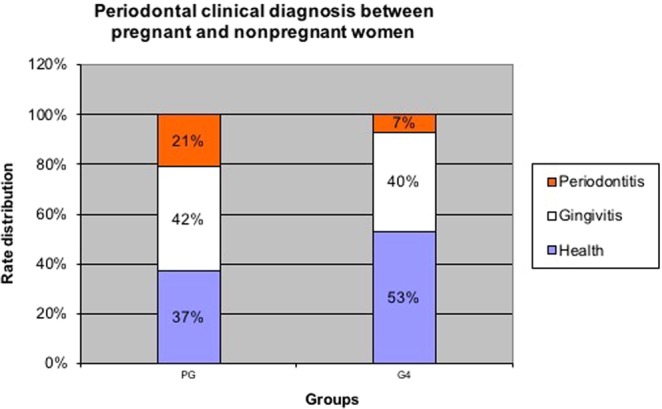
PG: pregnant group = all pregnant women of the sutdy (G1 + G2 + G3); G4 = nonpregnant group. This bar chart shows the rate distribution of periodontal clinical diagnosis when all pregnant women were compared to non-pregnant group.

Regarding the periodontal clinical diagnosis, no significant difference was observed between the groups (p = 0.1035, Chi-square test), and no association was noted between clinical diagnosis and epidemiological data (Table 2). Figure 1 shows percentual distribution of periodontal clinical diagnosis when all pregnant women combined were compared with nonpregnant.

**Table 2:** Comparison of periodontal clinical diagnosis among different groups (Chi-square test; p < 0,05).

Groups	Clinical Periodontal Diagnosis^a^								p-value
	Health		Gingivitis		Periodontitis		Total		
	N	%	N	%	N	%	N	%	
**1**	6	(37%)	10	(62%)	0	(0%)	16	(100%)	0.1035
**2**	7	(33%)	7	(33%)	7	(33%)	21	(100%)	
**3**	6	(40%)	5	(33%)	4	(27%)	15	(100%)	
**4**	8	(53%)	6	(40%)	1	(7%)	15	(100%)	

^a^The periodontal diagnosis was established after oral evaluation and was classified as healthy (when < 30% of the periodontal sites presented gingival bleeding), gingivitis (>30% of the periodontal sites presented gingival bleeding) or periodontitis (presence of four or more teeth with one or more sites presenting PD ≥ 4 mm and loss of clinical insertion ≥ 3 mm at the same site).

No significant variation was observed in the clinical parameters of PD (p = 0.546), CAL (p = 0.099), PI (p = 0.074) and GI (p = 0.217) by the ANOVA test, even when we combine all pregnant in one group (see Supplementary Table S1).

To evaluate differences in the total amount of bacteria, the ANOVA test was used, and no significant difference was observed between the groups (p = 0.4336). In contrast, when the periodontopathogenic microorganisms of interest were individually quantified, the detection of *Tf* revealed significant differences, with p = 0.013 (Table 3).

**Table 3:** Periodontopathogens quantification, total and by species, in the different groups (ANOVA test, p < 0.05).

Groups	Bacterias	Periodontopathogens^b^ Quantification					Total
		Pg	Aa	Tf	So	Un	
	n/N^a^	6/16	16/16	15/16	7/16	16/16	
**1**	Total Amount	8.991.856	1.436.886	3.237.496	585.962	63.252.639	77.504.839
	Mean counts	561.991	89.805	202.344	36.623	3.953.290	4.844.052
	Median	0	46.013	23.945	0	3.281.499	3.576.511
	Standard deviation	1.320.099	156.964	329.860	102.610	3.830.708	4.673.462
	n/N	11/21	21/21	17/21	9/21	21/21	
**2**	Total Amount	8.623.773	1.523.445	1.426.733	746.644	170.868.593	183.189.188
	Mean counts	410.656	72.545	67.940	35.554	2.999.282	3.585.977
	Median	40	18.709	1.147	0	1.429.628	1.443.286
	Standard deviation	1.244.766	138.342	133.984	79.832	3.661.119	5.063.654
	n/N	5/15	15/15	13/15	6/15	15/15	
**3**	Total Amount	2.021.614	897.862	472.465	277.626	35.799.713	39.469.280
	Mean counts	134.774	59.857	31.498	18.508	2.386.648	2.631.285
	Median	0	35.481	5.264	0	1.414.154	2.266.157
	Standard deviation	372.516	78.484	47.464	35.241	3.458.882	3.570.215
**4**	n/N	3/15	15/15	6/15	7/15	15/15	
	Total Amount	1.720.575	383.602	5.693	161.452	21.117.494	23.388.815
	Mean counts	114.705	25.573	380	10.763	1.407.833	1.559.254
	Median	0	24.561	0	0	917.895	956.179
	Standard deviation	422.722	22.010	483	19.993	1.261.805	1.501.765
**p-value**		0.515	0.467	0.013*	0.652	0.186	0.4336

^a^n/N: n = number of patients positive for each organism/N = number of patients in each group.

^b^Pg: Porphyromonas gingivalis; Aa: Aggregatibacter actinomycetemcomitans; Tf: Tannerella forsythia; So: Streptococcus oralis; Un: Universal; Total: all bacterias of interest.

*Significative p-value.

**Figure 2:**
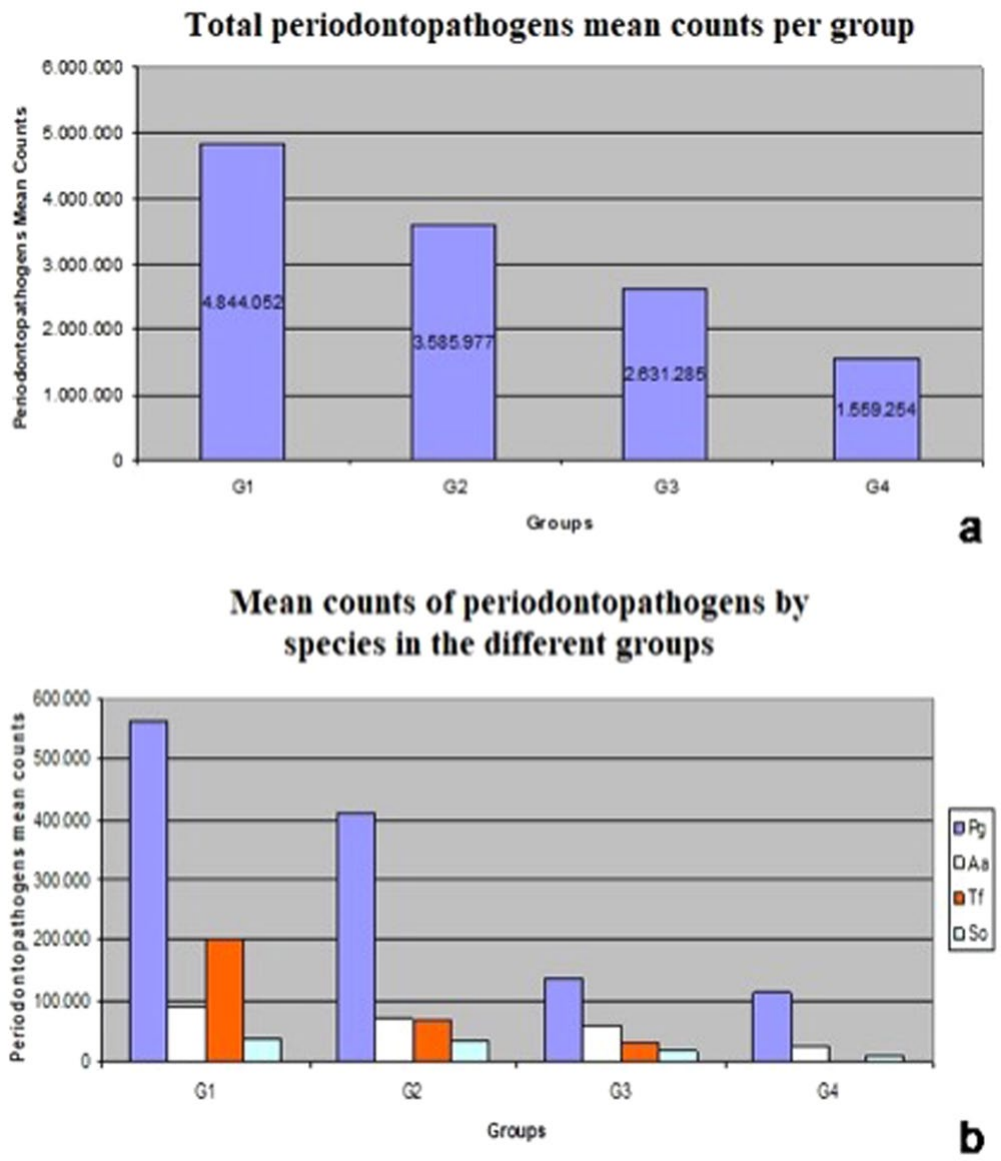
Periodontopathogens mean counts, total and by species. (**a**) Compares the total means of periodontopathogens in the different groups. (**b**) Compares mean counts of periodontopathogens by species in the different groups.

The Tukey’s multiple comparison test showed that there were differences in the quantification of *Tf* when comparing group 1 with groups 3 (p = 0.048) and 4 (p = 0.014). No significant difference was observed between groups 1 and 2 (p = 0.119). Figure 2 shows periodontopathogens mean counts, total and by species in different groups.

When we grouped all the pregnant women, also can be noticed that the total amount of subgingival bacteria had an influence on clinical diagnosis (p = 0.006), as shown in Table 4.

**Table 4:** Association between total bacterial quantification and periodontal clinical diagnosis among pregnant women (ANOVA test, p < 0.05).

Groups	Bacterial Quantification	Clinical Periodontal Diagnosis			p-value
		Health	Gingivitis	Periodontitis	
**1**	Mean counts	2.475.898	6.264.945	0	0.119
	Median	1.911.377	4.210.644	0	
	Standard deviation	1.874.674	5.335.504	0	
**2**	Mean counts	902.626	3.640.720	6.214.584	0.146
	Median	526.191	2.753.431	3.935.374	
	Standard deviation	845.962	2.373.493	7.915.453	
**3**	Mean counts	459.090	2.312.813	6.287.668	0.024*
	Median	216.069	2.316.411	3.936.126	
	Standard deviation	566.618	924.803	5.511.489	
**PG** ^**a**^	Mean counts	1.259.385	4.531.753	6.241.160	0.006*
	Median	648.389	3.353.085	3.935.374	
	Standard deviation	1.435.355	4.103.691	6.834.248	
**4**	Mean counts	1.402.892	1.903.534	744.473	0.736
	Median	889.354	1.142.871	744.473	
	Standard deviation	1.255.824	1.947.505	— ^b^	

^a^PG: pregnant group = all pregnant women of the study (G1 + G2 + G3).

^b^As only 1 patient was diagnosed with periodontitis, there is no standard deviation.

^*^Significative p-value.

Comparing patients with healthy periodontal diagnoses, with those who clinically presented disease (gingivitis or periodontitis), it was shown that the mean amounts of total subgingival bacteria observed in latter categories were progressively larger. In Tukey’s multiple comparison test, differences between healthy and gingivitis and healthy and periodontitis classifications were observed (p = 0.041 and 0.008, respectively).

In the specific quantification of periodontopathogenic bacteria, *Aa, Pg* and *So* showed no association with clinical diagnosis in any of the groups, but the amount of *Tf* positively correlated (p = 0.031) with the diagnosis of gingivitis among pregnant women (see Supplementary Table S2).

The relative frequency of periodontopathogenic bacteria studied was commonly higher than 50% in this population. *Aa* was present in all evaluated patients (100%) and Pg was present in more than half of the pregnant women in group 2 (52%). For *Tf*, detection rates among pregnant women were higher than 80%, reaching 88% in group 1, 81% in group 2, and 87% in group 3.

The levels of estradiol and progesterone were considerably lower in the control group compared to the other groups and reflected the progression of gestational age, increasing progressively from groups from 1 to 3. The ANOVA test did not show an association between serum estradiol and/or progesterone levels with clinical periodontal diagnosis or the total amount of bacteria. However, it was observed that in the first trimester, *Pg* was correlated with progesterone levels (p = 0.041), according to Table 5.

**Table 5:** Correlation of serum levels of estradiol and progesterone with bacterial quantification, by species, in different groups (Pearson’s correlation, p < 0.05).

Hormone	Groups	Correlation p-value	Bacterial Species				
			Un	Pg	Aa	Tf	So
**Estradiol**	**1**	Corr (r)^a^	−0.247	−0.113	−0.126	−0.162	0.002
		p-value	0.357	0.677	0.642	0.548	0.995
	**2**	Corr (r)	−0.184	−0.128	−0.189	−0.215	−0.163
		p-value	0.512	0.649	0.501	0.441	0.563
	**3**	Corr (r)	−0.255	−0.147	−0.299	−0.269	−0.205
		p-value	0.359	0.601	0.279	0.332	0.464
	**4**	Corr (r)	−0.056	−0.223	−0.060	0.193	−0.212
		p-value	0.842	0.423	0.832	0.490	0.449
**Progesterone**	**1**	Corr (r)	0.333	0.516	−0.124	0.391	−0.169
		p-value	0.208	0.041*	0.646	0.134	0.532
	**2**	Corr (r)	0.197	0.102	0.070	0.009	−0.137
		p-value	0.392	0.659	0.762	0.969	0.553
	**3**	Corr (r)	0.075	−0.248	−0.082	0.064	−0.014
		p-value	0.791	0.372	0.772	0.821	0.959
	**4**	Corr (r)	0.049	−0.010	0.010	0.363	−0.201
		p-value	0.863	0.970	0.971	0.184	0.472

^a^Corr (r) = correlation.

*Significative p-value.

## Discussion

Our study population demonstrated homogeneity for age, parity, race and socioeconomic level, which made the comparisons between the 4 groups valid. Furthermore, the sample size was similar to those of other studies on the same subject^4,6,22–26^. With respect to age, the mean was 24.67 years old (±1.26), compatible with those reported in other studies^4,6,27,28^. Patients identified as “high-risk pregnancies” were excluded, because several studies have linked periodontal disease with adverse obstetric outcomes^29–33^. Thus, avoiding those who presented conditions that could negatively influence gestation or who may already have modifications of subgingival flora (such diabetics, hypertensives, smokers and patients with an obstetric past) a selection bias was avoided.

As we did not have prior knowledge of the patient’s periodontal status, it was possible to evaluate the flora of healthy women and those presenting gingivitis and periodontitis, in agreement with the works of Kornman and Loesche, 1980^4^ and Carrillo-de-Albornoz *et al*.^6^ but differing from Usin *et al*.^28^, who chose to select patients who presented clinical signs of periodontal inflammation. This aspect allowed us to make a general evaluation that was not influenced by the presence of individuals with periodontal alterations, where the presence of more pathogenic microbiotas is assumed.

Regarding the technique adopted for bacterial quantification, our study has the advantage of having used qPCR, which allows for the identification and quantification of periodontopathogenic bacteria with greater sensitivity, differentiating our study from previous ones that used microbiological evaluations based on culture^4,6^ and conventional PCR methods^28^. To define the species of interest for this study, we have chosen agents that the current literature points out as being some of the most frequently related to development, progression and aggressiveness of periodontal disease. We verified that the selected bacteria are recurrent in several publications^7,28,31^, due to their clear involvement with the periodontal pathologies. Regarding the hormonal evaluation, we chose to assess the serum level dosage, since the crevicular fluid essentially represents a transudate of the plasma and presents very similar hormonal levels^34^.

Although several papers discuss pregnant periodontal status, we found that there were some gaps to be filled, in particular related to the rigor of periodontal examination. The use of four objectively measurable criteria for periodontal clinical evaluation (CAL, PD, PI and GI) differentiates this study from others. In previous studies^4,6,23,35,36^ an evaluation of the CAL was not included. However, according to Armitage (2000), cases of periodontitis need to be evaluated and stratified based on the loss of clinical insertion, since it is the parameter that has the highest specificity.

Regarding the periodontal clinical diagnosis, our investigation did not detect any variation between the groups (Table 2). The clinical condition was particularly equitably distributed in groups 2 (health = 7, gingivitis = 7, periodontitis = 7) and 3 (health = 6, gingivitis = 5, periodontitis = 4). Periodontal health was similar between the four groups. The individual evaluation of the clinical parameters GI, PI, PD and CAL indicated a tendency for an increased CAL in group 3 (p 0.099) and the PI in group 1 (p 0.074), although these differences did not rise to the level of significance. It should be noted that although no association was observed between gestation and periodontal changes, the observed value of p = 0.1035 reinforces that eventually, a larger sample size would show a difference.

About clinical parameters of periodontal evaluation, Kornman and Loesche, 1980^4^ describe a significant rise in GI between the first and second trimesters, with no change detected in the nonpregnant group during the study period. These authors reported that the PI remained unchanged throughout pregnancy and postpartum, and the same stability was observed in the different groups of the present study. However, in relation to GI, our findings are discordant, since no differences were observed among the 4 groups or when we regrouped the patients, classifying them as pregnant and nonpregnant (p = 0.124). Regarding this result, our data agree with the work of Jonson *et al*.^35^, who also did not observe significant variation during the progression of pregnancy or alterations in GI and PD when comparing pregnant and non-pregnant women.

Regarding the levels of the selected bacterial species of subgingival flora we observed that the literature reinforces the absence of significant changes during gestation, which is demonstrated in this study by the absence of observed differences among the groups in relation to the total bacterial quantification, which is in agreement with the previous studies of Löe and Silness^37^ Cohen *et al*.^38^ Kornman and Loesche^4^ Tilakaratne *et al*.^39^ and Novak *et al*.^40^. However, even with a similar bacterial level during different trimesters, many studies have reported increased gingivitis during the gestational period^4,6–8,10,23–25,41^, which was not observed in our study. This discrepancy may have been due to methodological differences and differences in clinical evaluation criteria.

Adriaens *et al*.^42^ reported that subgingival levels of bacteria associated with periodontitis did not change during pregnancy, nor at 4 to 6 weeks postpartum. Out of the 37 species, only 17 presented quantitative reduction, when comparing the first trimester and the postpartum period. No change occurred between the 12th and 28th weeks of pregnancy and the quantification of *Aa, Pg, Tf* and *T. denticola* did not change.

Among pregnant women with periodontitis, Novak *et al*.^40^, did not observe qualitative differences for the seven periodontopathogenic microorganisms studied. However, the literature indicates that specific qualitative changes occur, such as an increase of *Bacteroides melaninogenicus intermedius* in the second trimester^4^, a greater proportion of *Prevotella intermedia* in the first trimester and of *Pg* and *Aa* in the third trimester^6^.

The results of our study are in accordance with those described above, since no variation was observed in relation to the total number of bacteria observed in the different groups (p = 0.4336). However, variation in specific species was detected. The quantification of *Tf* revealed a significant difference in the subgingival biofilm (p = 0.013). Group 1 presented a much higher mean than that observed in group 3, and there was a numerical decrease in this bacterium with the progression of gestation. It is possible that changes in *Tf* during pregnancy were not detected in studies that used microbial culture because it is an anaerobic bacterium and is difficult to culture^43^, which was overcome in this study with the use of qPCR. Regarding the observed difference between groups 1 and 4, our data confirm the findings of Carrillo-de-Albornoz *et al*.^6^, which had already demonstrated a sharp decrease in the detection of *Tf* when evaluating women three months after delivery.

The socioeconomic level did not correlate with the total bacterial count, possibly because we worked with a SUS user population with a very homogeneous socioeconomic level, in which 72 and 19% of the patients belonged to classes D and E, respectively, making up 91% of the sample. In this study, a divergence from the literature regarding the observed high prevalence of *Tf* and Aa in a population with no comorbidities. According to Aas *et al*.^44^, in general, it is not possible to detect species that are typically associated with periodontitis and caries, which was not confirmed in our study. *Pg* was present in more than half of the pregnant women in their second trimester (52%), over an 80% prevalence of *Tf* was observed in all pregnant women and Aa was present in all evaluated patients (100%).

We believe that the use of qPCR has promoted an increase in our ability to identify such bacteria, but it is also possible that when addressing a low socioeconomic population, there is a real, even if subclinical, impairment of their periodontal health. Because they are young patients, it is possible that there was not sufficient time for more exuberant periodontal manifestations to be present.

Based on the concept that the presence of bacterial biofilms is the primary factor in the development of gingivitis and periodontal disease^3^, in this study we observed that the total amount of subgingival bacteria positively correlated with an overall worsening of clinical diagnosis in pregnant women (p = 0.006). So, a higher load of periodontopathogenic microorganisms, capable of supplanting host protection mechanisms, leads to the onset and progression of the disease. In addition, we noticed an association between the *Tf* count and the occurrence of gingivitis among pregnant women when considered together (p = 0.031).

We didn’t identify relation with the hormones levels but there are others hypothesis to explain greater total microbial level in pregnant women. Oral hygiene may be affected by typical nausea and vomiting of early pregnancy leading to bacteria accumulation and maybe gestational immunological adaption leads to reduction of protect factors, allowing an increase in bacteria total amount when compare with non-pregnant women.

Regarding the evaluation of hormonal levels, the observed dosage was justified due to the presence of receptors for the sex steroids in the gingival tissues. During gestation, the effects of elevated estrogens and progesterone on the gingival vasculature could explain an increased occurrence of edema, erythema, increased crevicular fluid, and bleeding. High levels of steroids are associated with increased vascular permeability in the gingival sulcus and possibly explain the exacerbation of crevicular fluid secretion in this situation^10,11^.

It is important to note that variations in the levels of sex steroids occur during and outside of the gestational period, and it is important that nonpregnant patients were always evaluated at the same stage of the menstrual cycle, in follicular phase, between the 7th and 13th days of the cycle in this study. The luteal phase has already been studied^4,6^ but we chose to evaluate the patients in the control group during the follicular phase of the menstrual cycle, since it exhibits a greater contrast in relation to the gestational period, especially due to the minimal influence of progesterone in this phase of the cycle.

Hugoson^11^ reported that the signs of gingivitis begin to manifest in the second month of gestation, worsening until the 8th month, with later regression occurring after the birth and was thus correlated with the hormonal levels. This pattern of behavior did not occur in this study, and we questioned the possibility that when we included the CAL in the clinical evaluation if some of the patients previously considered to have gingivitis were reclassified with the diagnosis of periodontitis, which may have caused the disagreement. After the statistical evaluation of the data, we did not detect an association between serum estradiol and/or progesterone levels with a clinical periodontal diagnosis or with the total amount of bacteria among the groups.

In the qualitative evaluation, *Pg* was observed to correlate with progesterone levels in group 1 (p = 0.041), which was also observed by Carrillo-de-Albornoz *et al*.^6^. It is believed that *Pg* favors a sudden elevation of progesterone levels in the first trimester of gestation^4^, using it with growth factor to replace vitamin K. The nonpersistence of this correlation during the second and third trimesters suggests saturation of metabolic pathways is involved. Similar behavior has been described for another periodontopathogenic bacillus by Carrillo-de-Albornoz *et al*.^5^ and Kornman and Loesche^4^ who observed positive correlations between sex steroid levels and presence of *Prevotella intermedia*, *Bacteroides melaninogenicus* ss. and *Fusobacterium nucleatum*, but these periodontopathogenic bacteria were not evaluated in our research. We identified a tendency for a positive correlation between bacterial quantification and estradiol level in group 2 (p = 0.093), but without reaching significance.

Quantitative differences were observed for *Tf* during gestation when comparing groups 1 and 3, but the higher prevalence of *Tf* among pregnant women from group 1 did not show a positive or negative correlation with hormonal levels, suggesting that their variation was not influenced by the studied steroids.

## Conclusions

In this study, the periodontal clinical diagnosis was positively correlated with the quantification of the subgingival microbiota during gestation. In the qualitative evaluation of the periodontopathogenic bacteria, we observed that *Tanerella forsithya* was more frequently observed among first-trimester pregnant women when compared to those in the third-trimester and nonpregnant women, with a decrease in their abundance observed during pregnancy. This periodontopathogenic bacterium, in turn, was associated with an increase in cases of gingivitis among pregnant women.

There was a positive correlation between serum progesterone levels and the presence of Pg, but no hormonal influence or epidemiological variables were identified for clinical diagnosis and total bacterial count. A high prevalence of known pathogenic bacteria was observed in this population of young women, without major current impairment of their clinical conditions, suggesting that preventive actions could prevent a future deterioration of their periodontal status.

## Supplementary information

Suplementary Tables

## Data Availability

All data generated or analysed during this study are included in this published article (and its Supplementary Information files).
