# Supplementary material for: Correlation of periodontal and microbiological evaluations, with serum levels of estradiol and progesterone, during different trimesters of gestation
Source: Sci Rep. 2019 Aug 13;9:11762. doi: 10.1038/s41598-019-48288-w (PMC6692383; doi:10.1038/s41598-019-48288-w)
Supplement: Supplementary file 1 — Suplementary Tables [file 41598_2019_48288_MOESM1_ESM.pdf]

Correlation of periodontal and microbiological evaluations, with serum levels of estradiol and progesterone, during different trimesters of gestation.

Renata Santos de Souza Massoni[1*], Andreza Maria Fábio Aranha[2], Fernanda Zanol Matos[2+], Orlando Aguirre Guedes[2+], Álvaro Henrique Borges[2+], Monize Miotto[2+], Alessandra Nogueira Porto[2+]

TABLES

TABLE S1 - Comparison of clinical periodontal parameters between pregnancy and not pregnant (ANOVA test, p <0.05).

Groups		Clinical Periodontal Parameters			
		PD	CIL	VPI	GBI
PG[a]	Mean counts	2.41	1.27	0.59	0.36
	Median	2.35	1.00	0,.58	0.22
	Standard deviation	0.61	0.74	0.35	0.34
G4[b]	Mean counts	2.33	1.00	0.49	0.22
	Median	2.30	1.00	0.50	0.26
	Standard deviation	0.58	0.74	0.31	0.17
p-value		0.624	0.223	0.311	0.124

[a]PG: pregnant group = all pregnant women of the study (G1 + G2 + G3);
[b]G4 = nonpregnant group.

TABLE S2 - Association between the amount of Tf and the periodontal clinical diagnosis (ANOVA test, p <0.05).

Groups	Bacterial Quantification	Clinical Periodontal Diagnosis			
		Health	Gingivitis	Periodontitis	Health
1	n/N[b]	6/16	10/16	0/16	0,079
	Mean counts	16.094	314.093	-	
	Median	124	117.005	-	
	Standard deviation	39.094	378.808	-	
2	n/N	7/21	7/21	7/21	0,454
	Mean counts	14.468	91.976	97.375	
	Median	1.175	1.027	1.024	
	Standard deviation	26.630	145.232	181.705	
3		6/15	5/15	4/15	0,073
	Mean counts	27	41.870	65.738	
	Median	30	61.592	37.468	
	Standard deviation	25	36.642	70.709	
PG[a]	n/N	19/52	22/52	11/52	0,031*
	Mean counts	10.421	181.551	85.871	
	Median	94	55.519	17.936	
	Standard deviation	26.721	288.904	146.850	
4	n/N	8/15	6/15	1/15	0,334
	Mean counts	246	456	986	
	Median	0	405	986	
	Standard deviation	456	503	-[c]	

[a]PG: pregnant group = all pregnant women of the study (G1 + G2 + G3)
[b]n/N: n = number of patients positive for each organism/N = number of patients in each group;
[C]As only 1 patient was diagnosed with periodontitis, there is no standard deviation.
*Significative p-value
